# The Beneficial Effects of Alpha-Blockers, Antimuscarinics, Beta 3-Agonist, and PDE5-Inhibitors for Ureteral Stent-Related Discomfort: A Systematic Review and Meta-Analysis from KSER Update Series

**DOI:** 10.3390/medicina61020232

**Published:** 2025-01-27

**Authors:** Young Joon Moon, Doo Yong Chung, Do Kyung Kim, Hae Do Jung, Seung Hyun Jeon, Seok Ho Kang, Sunghyun Paick, Joo Yong Lee

**Affiliations:** 1 Department of Urology, College of Medicine, Ewha Womans University, Seoul 07804, Republic of Korea; jjunny74@naver.com; 2 Department of Urology, Inha University College of Medicine, Incheon 22212, Republic of Korea; dychung@inha.ac.kr; 3 Department of Urology, Gangnam Severance Hospital, Urological Science Institute, Yonsei University College of Medicine, Seoul 03722, Republic of Korea; dokyung80@yuhs.ac; 4 Department of Urology, Inje University Ilsan Paik Hospital, College of Medicine, Inje University, Goyang 10380, Republic of Korea; haedojung@paik.ac.kr; 5 Department of Urology, Kyung Hee University School of Medicine, Seoul 02447, Republic of Korea; juro@khu.ac.kr; 6 Department of Urology, Korea University College of Medicine, Seoul 02841, Republic of Korea; mdksh@korea.ac.kr; 7 Department of Urology, Konkuk University School of Medicine, Seoul 05030, Republic of Korea; 8 Department of Urology, Severance Hospital, Urological Science Institute, Yonsei University College of Medicine, Seoul 03722, Republic of Korea; 9 Division of Medical Device, Clinical Trials Center, Severance Hospital, Yonsei University Health System, Seoul 03722, Republic of Korea

**Keywords:** network meta-analysis, stents, urolithiasis

## Abstract

*Background and Objectives*: Ureteral stents are widely used in the field of urology but can cause varying degrees of side effects. This study utilized a network meta-analysis to evaluate stent-related discomfort (SRD) in patients with alpha-blockers (alfuzosin, tamsulosin, and silodosin), antimuscarinics (solifenacin), beta 3-agonists (mirabegron), and phosphodiesterase 5-inhibitors (tadalafil) versus a placebo. *Materials and Methods*: Relevant randomized controlled trials (RCTs) from 2006 to 2021 were identified from electronic databases, including PubMed, EMBASE, and the Cochrane Library. The following identifiers were included to assess the urinary symptom score (USS): participants (patients with ureteral stents), interventions (patients who took medication for stent discomfort), and outcomes (comparisons of the Ureteric Stent Symptoms Questionnaire (USSQ)). We also executed an independent quality assessment using the Scottish Intercollegiate Guidelines Network (SIGN). *Results*: A total of 16 RCTs were identified, and they included 1865 patients. Compared with the placebo, mirabegron (mean difference (MD): −3.87; 95% confidence interval (CI): −10.6–2.35), tadalafil (MD: −4.47; 95% CI: −10.8–1.63), and silodosin (MD: −4.02; 95% CI: −12–4.01) did not show significant differences to the placebo, whereas others did. Alfuzosin, mirabegron, silodosin, solifenacin, and tadalafil were not inferior to tamsulosin in terms of the USS using Bayesian analyses. In the random effect model, P-score tests showed that solifenacin possessed the highest P-score (*p* = 0.8484); tamsulosin was the second highest (*p* = 0.7054). As a result of the rank-probability test, solifenacin was also ranked highest in terms of USS, and tamsulosin was ranked second. *Conclusions*: Compared with the placebo, solifenacin, tamsulosin, and alfuzosin significantly decreased the USS. In our study, solifenacin may be considered the most effective medication for SRD.

## 1. Introduction

Ureteral stents are widely used in the field of urology to facilitate the flow of urine in the urinary tract or to help restore and stabilize the ureteral anastomosis site [1]. Clinically, indwelling of the ureteral stent is recommended to drain urine in patients with obstructive uropathy [2]. Ureteral stents are widely used in the field of urology but can cause varying degrees of side effects: pain that affects daily life, urinary symptoms, reduced work efficiency, and reduced quality of life [3]. After cystoscopic ureteral stent insertion was reported in 1967 by Zimskind et al. [4], various studies have been conducted for a long time to manage stent-related discomfort (SRD). Several drugs have been studied and used to reduce the incidence of SRD after indwelling ureteral stents. The main medications used to alleviate SRD are alpha-blockers and antimuscarinic drugs [5]. The American Urological Association (AUA)/Society of Endourology Guidelines suggests alpha-blockers and antimuscarinic therapies to reduce SRD as a moderate recommendation with level B evidence [6]. The European Association of Urology (EAU) guidelines recommend only alpha-blockers as medications to reduce SRD (strong recommendation with level 1a evidence) [7]. Although the exact mechanism of SRD is unknown, there are reports that smooth muscle spasms may be related to the occurrence of SRD [8]. Beta-3 agonists are effective treatments for overactive bladder syndrome and have been reported to reduce bladder contractions, urgency, and urinary frequency [9]. Additionally, several studies have described relief of lower urinary tract symptoms (LUTS) in patients treated for erectile dysfunction (ED) with PDE5 inhibitors [10]. Based on these reports, studies on other drugs to alleviate SRD, such as beta 3-agonists, phosphodiesterase-5 inhibitors (PDE5-inhibitor), and their combinations, have been conducted and reported.

We conducted a network meta-analysis to evaluate SRD in patients with alpha-blockers (alfuzosin, tamsulosin, and silodosin), antimuscarinics (solifenacin), beta 3-agonists (mirabegron), and PDE5-inhibitors (tadalafil) versus a placebo.

## 2. Materials and Methods

### 2.1. Inclusion Criteria

The eligibility of a study was evaluated by considering participants, interventions, comparators, outcomes, study design (PICOS) approach, and the Preferred Reporting Items for Systematic Reviews and Meta-Analyses (PRISMA) guidelines (Table S1. PRISMA Checklist).

Patients were those with ureteral stents. The intervention was medication for stent discomfort. The outcomes were comparisons of the Ureteric Stent Symptoms Questionnaire (USSQ) to assess the urinary symptom score (USS).

### 2.2. Search Strategy

A literature search of all publications from 2006 to 2021 was performed using PubMed, EMBASE, and the Cochrane Library. Information about the proceedings of relevant meetings was also searched for. Combinations of the following medical subject headings terms and keywords were used: ureteral stent, ureteral stent-related discomfort, ureteral stent-related symptoms, alpha-blocker, antimuscarinics, beta 3-agonist, and PDE5-inhibitor.

### 2.3. Data Extraction

Two researchers (YJM and HDJ) screened all retrieved titles and abstracts according to the search strategy. Two other researchers (DYC and DHK) independently evaluated the full article text to search for further relevant articles. Data were recorded, including the author, publication year, country, study design, and procedure, from the most relevant articles. Disagreements were resolved by conducting discussions until consensus was reached or through mediation by another researcher (JYL).

### 2.4. Quality Assessment for Studies

We used the Cochrane Risk of Bias tool for randomized controlled trials (RCTs). We graded the quality of evidence for all eligible studies using the Scottish Intercollegiate Guidelines Network (SIGN). Our researchers carried out a quality assessment independently (YJM and HDJ). Differences in opinion regarding quality assessment results were resolved by discussion with a third reviewer (JYL). Quality assessment was performed with Review Manager 5 (RevMan 5.4.1, Cochrane Collaboration, Oxford, UK).

### 2.5. Heterogeneity Tests

We assessed the presence of heterogeneity using the Q and Higgins’ I^2^ statistics [11]. The Q statistic was used to evaluate statistical heterogeneity, whereas I^2^ was used to quantify heterogeneity. Higgins’ I^2^ measures the percentage of total variation due to heterogeneity rather than chance across studies. Higgins’ I^2^ was calculated as follows:

$$I^{2}=\frac{Q-\mathrm{df}}{Q}\times 100\%$$

where “Q” is Cochran’s heterogeneity statistic and “df” is the degrees of freedom. An I^2^ ≥ 50% was considered substantial heterogeneity. For the Q statistic, heterogeneity was deemed significant for *p* < 0.10 [12]. If I^2^ < 50%, we applied the fixed effect model; otherwise, the random effect model was applied. Studies with confirmed positive results used a pooled specificity with 95% confidence intervals (CIs). Additionally, Galbraith radial plots evaluated heterogeneity [13,14].

### 2.6. Statistical Analysis

Using a network meta-analysis, we measured the treatment effect for dichotomous outcomes using the mean difference (MD) with a 95% CI. A sensitivity analysis determined whether the heterogeneity resulted from low study quality. The results of the meta-analysis are presented in forest plots. Publication bias was assessed with a funnel plot. All statistical analyses were performed through R software (version 4.1.2, R Foundation for Statistical Computing, Vienna, Austria; http://www.r-project.org (accessed on 10 April 2021)) and with the associated meta, netmeta, pcnetmeta, and gemtc packages for pairwise and network meta-analyses [15]. This systematic review is registered in PROSPERO, CRD42022336914.

## 3. Results

### 3.1. Eligible Studies

**Figure 1:**
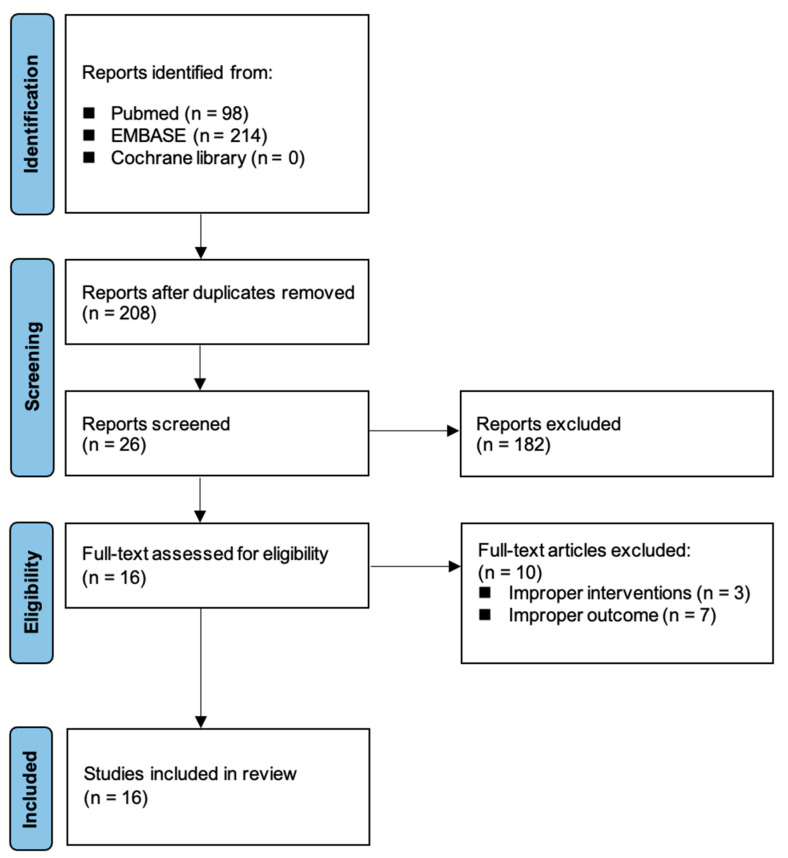
Study flow chart.

The initial database search verified a total of 312 articles. Of these, 296 articles were excluded: 88 were duplicate publications, and 182 were excluded after abstract review. A total of 26 articles were selected for full-text evaluation. Further review excluded 10 articles as they were irrelevant to the analysis: 3 for cited improper interventions and 7 for improper outcomes. Finally, 16 RCTs, including 1865 patients, were identified as relevant for the current study and selected for the meta-analysis [10,16,17,18,19,20,21,22,23,24,25,26,27,28,29,30]. Figure 1 shows the study flow chart.

### 3.2. Characteristics of the Included Studies

Table 1 describes the characteristics of all included studies [10,16,17,18,19,20,21,22,23,24,25,26,27,28,29,30]. The studies were published from January 2006 to January 2021. Eight of the sixteen studies were performed in Asia (Iran, South Korea, India, Pakistan, and Taiwan) [10,17,18,22,23,25,26,27], three in Egypt [19,20,21], two in Greece [24,30], one in the USA [28], one in Italy [29], and one in Turkey [16].

**Figure 2:**
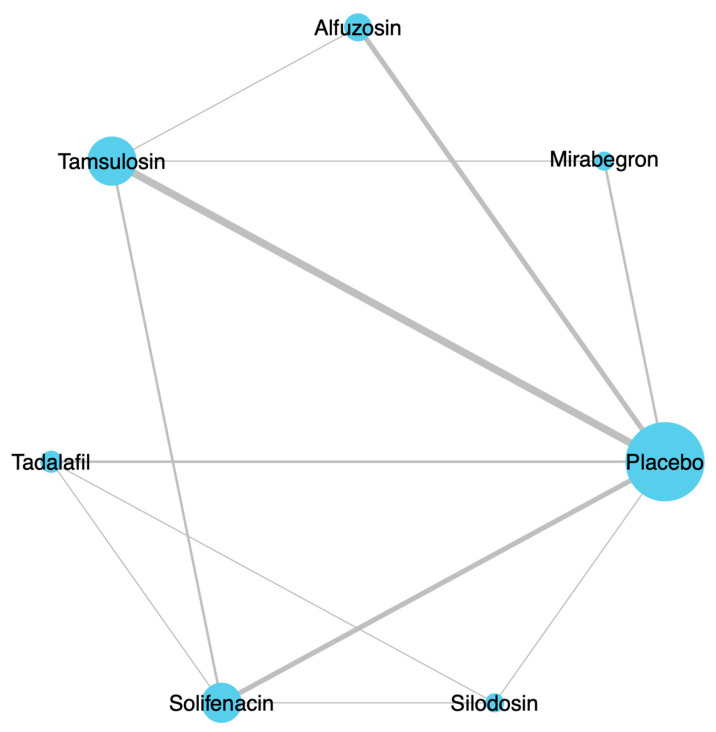
Network plots for included studies. A total of 16 RCT articles were identified, including 1865 patients. RCT; randomized controlled trial.

Five studies compared alfuzosin and a placebo [24,25,26,28,30]. Eight trials compared tamsulosin and a placebo [16,20,21,22,23,24,27,29]. One study compared silodosin and a placebo [18]. Five studies compared solifenacin and a placebo [18,19,20,21,22]. Two articles compared mirabegron and a placebo [16,17]. Two studies compared tadalafil and a placebo [10,18] (Figure 2).

### 3.3. Quality Assessment and Publication Bias

**Figure 3:**
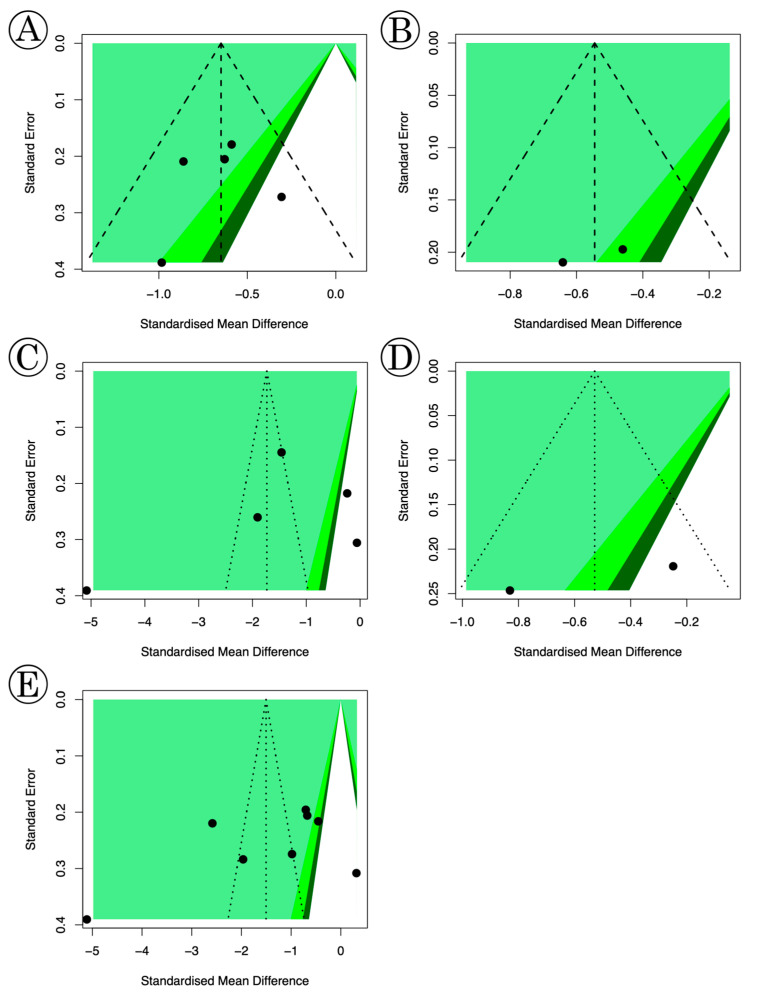
Funnel plots of (**A**) alfuzosin, (**B**) mirabegron, (**C**) solifenacin, (**D**) tadalafil, and (**E**) tamsulosin. There was little publication bias in the funnel plots.

**Figure 4:**
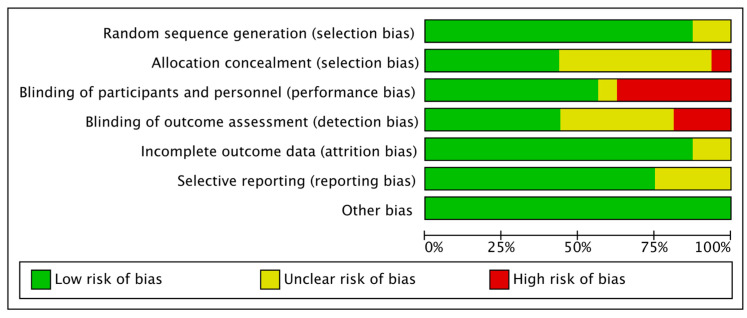
Risk of bias for eight RCTs. The risk of bias for each item is presented as a percent across all included studies. RCT: randomized controlled trial.

**Figure 5:**
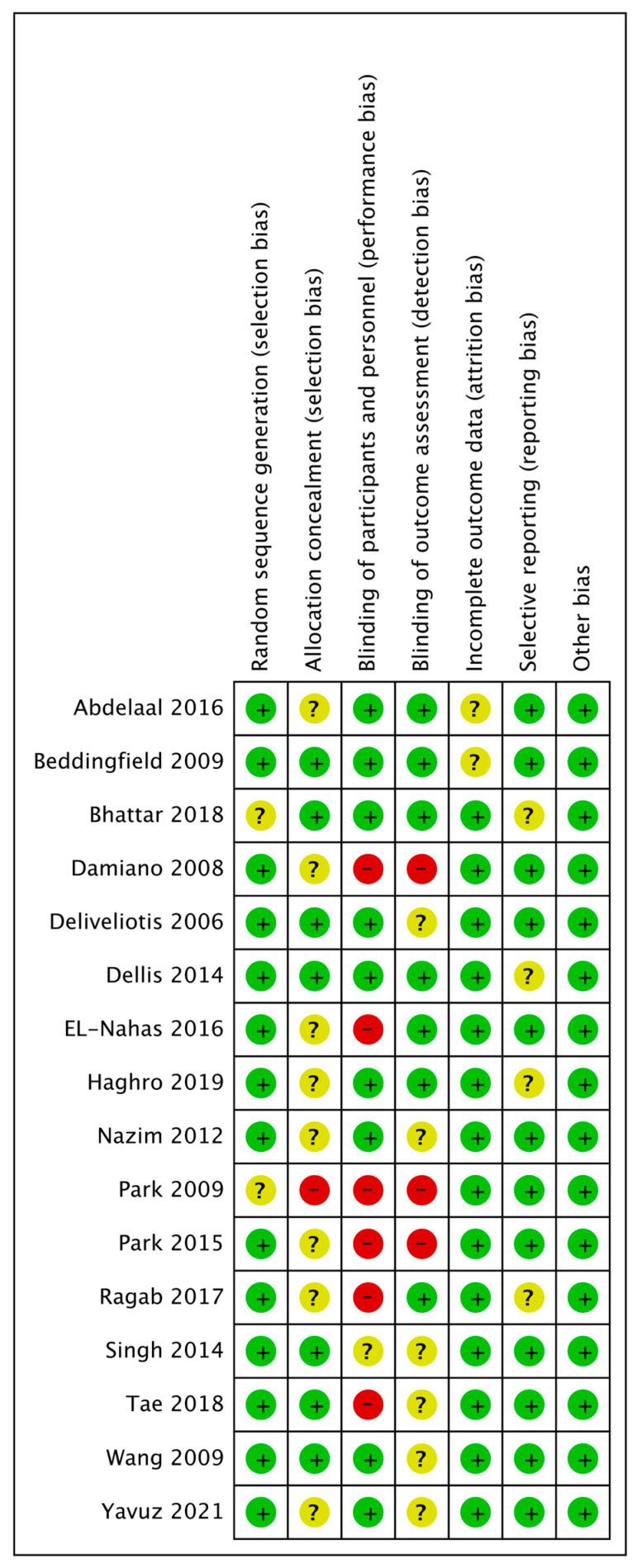
Risk of bias for eight RCTs. +, no bias; –, bias; ?, bias unknown. RCT: randomized controlled trial [10,16,17,18,19,20,21,22,23,24,25,26,27,28,29,30].

The quality assessment results using the SIGN are provided in Table 1 and were found acceptable. Using the SIGN checklist, seven studies were rated as 1+, six as 2+, and three as 1−. Funnel plots of our study are shown in Figure 3, and little publication bias was observed. The risk of bias for the RCTs included in this study is shown in Figure 4 and Figure 5. All studies were considered appropriate.

### 3.4. Heterogeneity and Inconsistency Assessment

**Figure 6:**
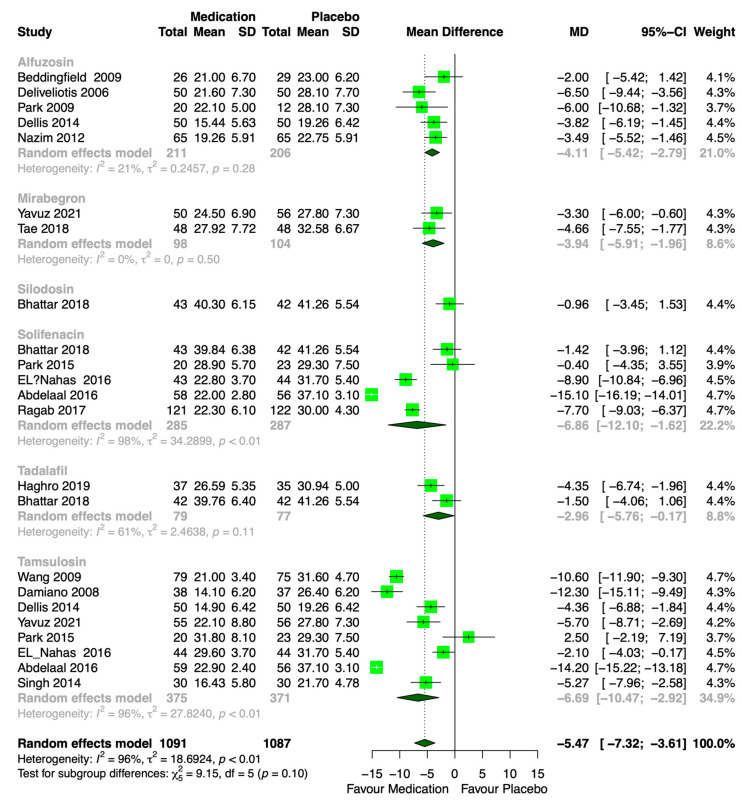
Pairwise meta-analysis of alfuzosin, mirabegron, silodosin, solifenacin, tadalafil, and tamsulosin. The USS of alfuzosin, mirabegron, silodosin, solifenacin, tadalafil, and tamsulosin was lower than that of the placebo. USS: urinary symptom score [10,16,17,18,19,20,21,22,23,24,25,26,27,28,29,30].

**Figure 7:**
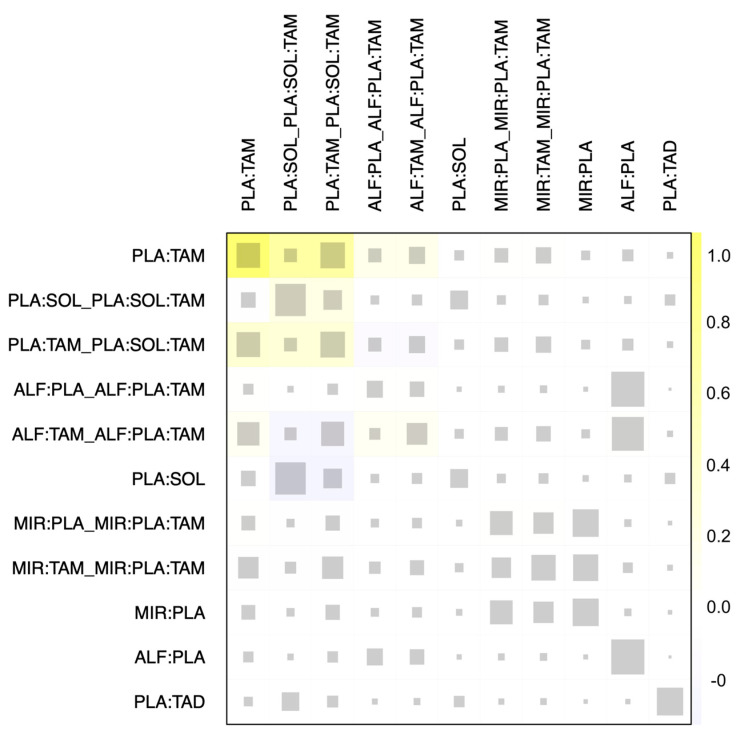
Net heat plot for inconsistency. Net heat plot shows little inconsistency within the whole network. PLA, placebo; ALF, alfuzosin; MIR, mirabegron; SOL, solifenacin; TAD, tadalafil; TAM, tamsulosin.

Forest plots of the pairwise meta-analysis results of alfuzosin, mirabegron, silodosin, solifenacin, tadalafil, and tamsulosin are shown in Figure 6. No heterogeneity was found between the placebo and alfuzosin or mirabegron in any study; however, some heterogeneity was encountered between the placebo and solifenacin, tadalafil, or tamsulosin. A net heat plot also revealed some inconsistency in the whole network (Figure 7).

### 3.5. Pairwise Meta-Analysis of Each Medication Compared with Placebo

A pairwise meta-analysis of the USS between each medication and the placebo is shown in Figure 6. The pooled data that compared the USS between alfuzosin and the placebo showed a significantly lower alfuzosin USS (MD: −4.11: 95% CI: −5.42 to −2.79, *p* < 0.001). The mirabegron USS was lower than that of the placebo (MD: −3.94, 95% CI: −5.91 to −1.96, *p* < 0.001). The solifenacin USS was lower than that of the placebo (MD: −6.86, 95% CI: −12.10 to −1.62, *p* < 0.001). The tadalafil USS was lower than that of the placebo (MD: −2.96, 95% CI: −5.76 to −0.17, *p* < 0.001). And last, the tamsulosin USS was lower than that of the placebo (MD: −6.69; 95% CI: −10.47 to −2.92, *p* < 0.001).

### 3.6. Network Meta-Analysis of Each Medication for USS

**Figure 8:**
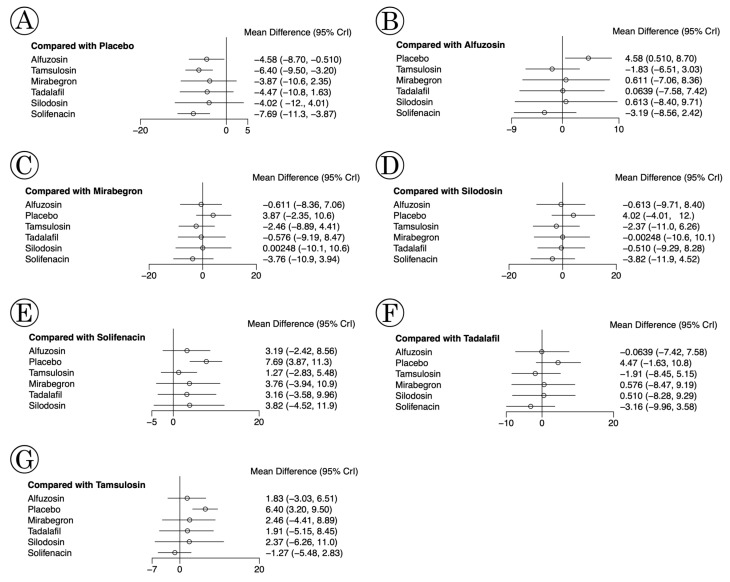
Network meta-analysis of each medication for USS; Compared with (**A**) placebo, (**B**) alfuzosin, (**C**) mirabegron, (**D**) silodosin, (**E**) solifenacin, (**F**) tadalafil, and (**G**) tamsulosin. Compared with the placebo, mirabegron (MD: −3.87, 95% CI: −10.6 to 2.35), tadalafil (MD: −4.47, 95% CI: −10.8 to 1.63), and silodosin (MD: −4.02, 95% CI: −12 to 4.01) did not show significant differences, whereas others did. All medications had insignificant differences, except for the placebo, compared with solifenacin. Alfuzosin, mirabegron, silodosin, solifenacin, and tadalafil were not inferior to tamsulosin in the USS using Bayesian analyses. MD, mean difference; USS, urinary symptom score; CI, confidence interval.

**Figure 9:**
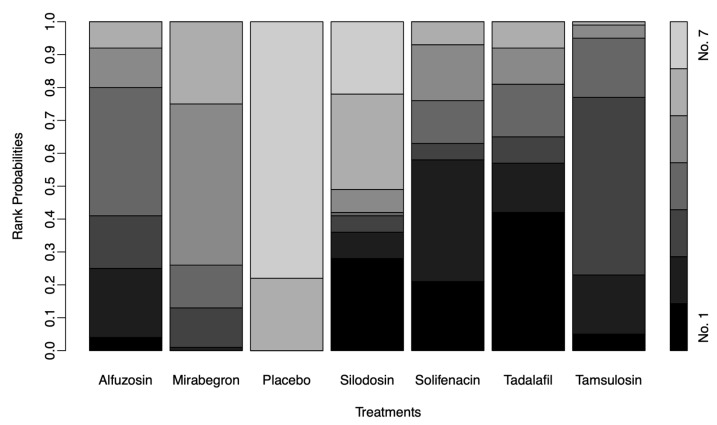
The rank-probability test. In the rankogram, solifenacin also had the highest rank for USS, followed by tamsulosin. USS, urinary symptom score.

In the network meta-analyses, the USS of mirabegron (MD: −3.87, 95% CI: −10.6 to 2.35), tadalafil (MD: −4.47, 95% CI: −10.8 to 1.63), and silodosin (MD: −4.02, 95% CI: −12 to 4.01) did not exhibit significant differences to the placebo, whereas others did (Figure 8). Compared with solifenacin, all medications possessed insignificant differences, except for the placebo (MD: 7.69, 95% CI: 3.87 to 11.3). When comparing tamsulosin, alfuzosin, mirabegron, and silodosin using Bayesian analyses of the USS, solifenacin and tadalafil were not inferior to tamsulosin. In the random effect model, P-score tests using frequentist inference to rank treatments in the network showed that solifenacin possessed the highest P-score (*p* = 0.8484); tamsulosin was the second highest (*p* = 0.7054). As a result of the rank-probability test, solifenacin was also ranked highest in USS, and tamsulosin was ranked second (Figure 9).

## 4. Discussion

Ureteral stents are useful and commonly used clinical tools in the urologic field. However, up to 88% of patients with ureteral stents suffer from SRD, and over 70% of these patients require the use of analgesics [5]. Regarding SRD management, there are some differences in recommendations for each guideline. The AUA/Society of Endourology guidelines suggest alpha-blockers and antimuscarinic therapies to reduce SRD as a moderate recommendation with level B evidence [6]. However, the EAU guidelines recommend only alpha-blockers as medications to reduce SRD (strong recommendation with level 1a evidence) [7]. Additional studies on the medical treatment of SRD are needed to establish treatment guidelines for effective SRD management. The object of this study was to evaluate SRD in patients with alpha-blockers (alfuzosin, tamsulosin, and silodosin), antimuscarinics (solifenacin), beta 3-agonists (mirabegron), and PDE5-inhibitors (tadalafil) versus a placebo.

According to the results of our study, in the network meta-analyses, the USSs of alfuzosin, tamsulosin, and solifenacin exhibit significant differences to the placebo. And, in the rank-probability test, solifenacin was also ranked highest in USS, and tamsulosin was ranked second. Several systematic reviews and meta-analysis studies compared the effects of alpha-blockers and antimuscarinics on ureteral stent-related discomfort. Kwon et al. [31] conducted a network meta-analysis to evaluate SRD in patients with alfuzosin or tamsulosin versus a placebo. In the network meta-analysis, both alfuzosin and tamsulosin showed lower scores compared with the placebo. As a result of the rank-probability test, tamsulosin was ranked highest in USS and BPS, and alfuzosin was ranked second. Alpha-blockers statistically significantly reduced the USS and BPS compared to the placebo, and tamsulosin may be more effective than alfuzosin. It is still unclear whether subtype selectivity is an important factor influencing differences in the efficacy of alpha-blockers. The authors proposed the hypothesis that tamsulosin, a selective alpha-1a and alpha-1d blocker, showed higher efficacy than alfuzosin, a non-selective alpha-1 blocker, due to the high distribution of alpha-1d receptors in the distal ureter. Although additional study is needed in the future, this subtype selectivity may also be an important consideration in drug selection in SRD management.

Gao et al. conducted a meta-analysis comparing alpha-blockers and antimuscarinic monotherapies in releasing ureteral stent-related symptoms [32]. USSQ scores were statistically significantly lower in the alpha-blocker group. However, there were no significant differences in urinary symptoms, pain, general health, work performance, and sexual performance between the two groups. They concluded that alpha-blockers showed a similar effect with antimuscarinics.

Nonsteroidal anti-inflammatory drugs (NSAIDs) have also been studied for their effectiveness in reducing SRD. Tadros et al. [33] conducted RCT of a single dose of rofecoxib 50 mg or placebo before ureteral stent removal. A total of 22 patients were enrolled, and pain was assessed by VAS score just before and 24 h after stent removal. The proportion of patients who complained of severe pain with a VAS score of 7 or higher for 24 h after ureteral stent removal was statistically significantly higher in the placebo group at 55% (6/11) than in the NSAIDs group at 0% (0/11) (*p* < 0.01). They concluded that a single dose of a NSAIDs before stent removal is a simple and cost-effective method for preventing severe pain after ureteric stent removal.

Despite the high prevalence of SRD, the definite cause of SRD is still unknown. The mechanism of SRD may be associated trigone irritation, smooth muscle spasm, or a combination of factors [8]. Studies have shown that alpha-blockers are more effective for pain than urinary symptoms [34]. Based on these results, the authors proposed the hypothesis that smooth muscle spasms of the ureter and/or bladder play an important role in the occurrence of SRD. In contrast, the results of a study that reported looped stents are not effective in improving SRD suggest that trigone contact is not as significant as once thought [35]. SRD may occur secondary to urine reflux through the stent during voiding [5]. Intravesical pressure also increases with detrusor contraction, which transfers this increased pressure to the renal collecting system, ultimately causing flank pain. Physical activity and detrusor contraction cause movement of the stent, and this movement may be an additional cause of pain and urinary symptoms through direct stimulation of the bladder mucosa.

Various stent characteristics including material, diameter, length, and shape have been changed to decrease SRD. Several studies have compared stent-related discomfort between softer ureteral stents and stiffer stents, with different results. Lennon et al. conducted a randomized study of 155 patients to compare biocompatibility and patient tolerance between firm polyurethane and soft hydrophilic polymer stents [36]. The incidence of dysuria with renal and suprapubic pain was significantly higher in the firm stent group. However, there was no significant difference in voiding symptoms such as urgency, frequency, and nocturia and hematuria. In another study, Joshi et al. [37] conducted a study comparing the difference in SRD between firm and soft polymer stents in 116 patients with urinary tract stones who required insertion of ureteral stents. The two stents are made by the same company and have similar structural and functional properties. In the results of the study, there was no difference between the two groups in the effect on the patient’s health-related quality of life. Therefore, they concluded that stent composition alone does not affect patient quality of life.

Erturk et al. [38] conducted a study to compare SRD according to stent diameters (4.7 Fr. vs. 6 Fr.) in a total of 46 patients who were diagnosed with urinary stones and underwent ureteroscopy. Stent-related pain and irritation symptoms were evaluated through a questionnaire with a minimum score of 0 (none) and a maximum score of 5 (severe). There was no statistically significant difference in pain and irritation symptoms between the two groups. However, there was more distal migration and dislodgement of the stent in the 4.7 Fr. stent group than in the 6 Fr. stent group. In conclusion, the authors recommended the use of a stent of at least 6 Fr. when stent insertion is necessary after ureteroscopy.

There was also a study on SRD depending on stent length. Ho et al. [39] conducted a study comparing the differences in SRD according to stent length (22, 24, or 26 cm) in a total of 87 patients who underwent ureteroscopic surgery for ureteral stones. SRD was assessed through a questionnaire. The use of longer stents was statistically significantly associated with a higher incidence and greater severity of urinary frequency (*p* = 0.04 and *p* < 0.01, respectively) and urgency (*p* = 0.02, and *p* < 0.01, respectively). However, there was no statistically significant difference in flank pain between the three groups. The authors concluded that the use of longer stents was associated with a higher incidence and more severe urinary symptoms. The authors stated that the reason for this was that when a longer stent was used, the intravesical segment of the stent became longer and the irritation symptoms became worse. Additionally, based on the study results, they recommended that the use of a 22 cm long stent is more appropriate in patients with a height between 149.5 cm and 178.5 cm (median 161.9 cm).

Though the included studies met quality standards, our study has some limitations. First, we did not evaluate differences in efficacy by specific type of alpha-blocker, antimuscarinic agent, or PDE5-inhibitor. In addition, bias may have been introduced by evaluating studies with different medication doses and outcome assessment times and with inconsistent/unavailable safety data recordings. Second, other methods of evaluating clinical efficacy, such as the International Prostate Symptom Score (IPSS), quality of life (QoL), and visual analogue pain scale (VAPS), were not analyzed. Third, the side effects of the drugs and their suitability according to their severity were not analyzed. The type and severity of side effects are important considerations in the selection of therapeutic agents for the management of SRD. An additional limitation was not considering the possible effects of stent factors, such as stent sizes and materials used in the procedures on stent discomfort. Publication bias that may occur in network meta-analysis studies was also considered a limitation of this study [40]. We anticipate that further study of the various limitations of our study will increase the level of evidence for our results.

## 5. Conclusions

Solifenacin, tamsulosin, and alfuzosin significantly decreased the USS compared with the placebo. Solifenacin may be more effective than other drugs in urinary symptom control. Tamsulosin may be considered the second most effective medication for stent-related discomfort.

These results, along with others from further studies on combination therapy, may be helpful for urologists in selecting drugs for the management of stent-related discomfort.

## Figures and Tables

**Table 1:** Characteristics of included studies.

Author (Year)	Country	Study Design	Medication	No. Patients	USS Mean	USS SD	Quality Assessment (SIGN) ^a^
Yavuz et al., 2021 [16]	Turkey	RCT	Mirabegron 50 mg	50	24.5	6.9	1+
			Tamsulosin 0.4 mg	55	22.1	8.8	
			Control	56	27.8	7.3	
Haghro et al., 2019 [10]	Iran	RCT	Tadalafil	37	26.59	5.35	1+
			Control	35	30.94	5	
Tae et al., 2018 [17]	South Korea	RCT	Mirabegron 50 mg	48	27.92	7.72	2+
			Control	48	32.58	6.67	
Bhattar et al., 2018 [18]	India	RCT	Silodosin	43	40.3	6.151	1+
			Solifenacin 5 mg	43	39.84	6.384	
			Tadalafil	42	39.76	6.404	
			Control	42	41.26	5.539	
Ragab et al., 2017 [19]	Egypt	RCT	Solifenacin 5 mg	121	22.30	6.1	1+
			Control	122	30.00	4.3	
EL-Nahas et al., 2016 [20]	Egypt	RCT	Tamsulosin 0.4 mg	44	29.6	3.7	1+
			Solifenacin 5 mg	43	22.8	3.7	
			Control	44	31.7	5.4	
Abdelaal et al., 2016 [21]	Egypt	RCT	Tamsulosin 0.4 mg	59	22.9	2.4	1+
			Solifenacin 5 mg	58	22	2.8	
			Control	56	37.1	3.1	
Park et al., 2015 [22]	South Korea	RCT	Tamsulosin 0.4 mg	20	31.8	8.1	2+
			Solifenacin 5 mg	20	28.9	5.7	
			Control	23	29.3	7.5	
Singh et al., 2014 [23]	India	RCT	Tamsulosin 0.4 mg	30	16.43	5.8	1−
			Control	30	21.7	4.78	
Dellis et al., 2014 [24]	Greece	RCT	Tamsulosin 0.4 mg	50	14.9	6.425	2+
			Alfuzosin 10 mg	50	15.44	5.63	
			Control	50	19.26	6.425	
Nazim et al., 2012 [25]	Pakistan	RCT	Alfuzosin 10 mg	65	19.26	5.9061087	1−
			Control	65	22.75	5.9061087	
Park et al., 2009 [26]	South Korea	RCT	Alfuzosin 10 mg	20	22.1	5	1−
			Control	12	28.1	7.3	
Wang et al., 2009 [27]	Taiwan	RCT	Tamsulosin 0.4 mg	79	21	3.4	2+
			Control	75	31.6	4.7	
Beddingfield et al., 2009 [28]	USA	RCT	Alfuzosin 10 mg	26	21	6.7	2+
			Control	29	23	6.2	
Damiano et al., 2008 [29]	Italy	RCT	Tamsulosin 0.4 mg	38	14.1	6.2	1+
			Control	37	26.4	6.2	
Deliveliotis et al., 2006 [30]	Greece	RCT	Alfuzosin 10 mg	50	21.6	7.3	2+
			Control	50	28.1	7.7	

RCT: randomized controlled trial; USS: urinary symptom score; SD: standard deviation. ^a^ The quality assessment was indicated by the Scottish Intercollegiate Guidelines Network (SIGN) checklist. The symbol 1+ indicates a well-conducted RCT with a low risk of bias, 1− indicates RCTs with a high risk of bias, 2+ indicates well-conducted cohort studies with a low risk of bias.

## Data Availability

The data presented in this study are available in the article.

## Supplementary Materials

The following supporting information can be downloaded at: https://www.mdpi.com/article/10.3390/medicina61020232/s1, Table S1: PRISMA Checklist [41].
